# Bloodstream infection clusters for critically ill patients: analysis of two-center retrospective cohorts

**DOI:** 10.1186/s12879-024-09203-5

**Published:** 2024-03-13

**Authors:** Lei Wang, Li Zhang, Xiaolong Huang, Hao Xu, Wei Huang

**Affiliations:** 1 Department of Critical Care Medicine, The First Hospital of Shanxi Medical University, Taiyuan, China; 2 Department of Critical Care Medicine, The First Affiliated Hospital of Xiamen University, School of Medicine, Xiamen University, Xiamen, China; 3 The Third Clinical Medical College, Fujian Medical University, Fuzhou, China

**Keywords:** Bloodstream infection, Critically ill patients, Prediction score, Prognosis

## Abstract

**Background:**

Bloodstream infections (BSI) are highly prevalent in hospitalized patients requiring intensive care. They are among the most serious infections and are highly associated with sepsis or septic shock, which can lead to prolonged hospital stays and high healthcare costs. This study aimed at establishing an easy-to-use nomogram for predicting the prognosis of patients with BSI.

**Methods:**

In retrospective study, records of patients with BSI admitted to the intensive care unit (ICU) over the period from Jan 1st 2016 to Dec 31st 2021 were included. We used data from two different China hospitals as development cohort and validation cohort respectively. The demographic and clinical data of patients were collected. Based on all baseline data, k-means algorithm was applied to discover the groups of BSI phenotypes with different prognostic outcomes, which was confirmed by Kaplan-Meier analysis and compared using log-rank tests. Univariate Cox regression analyses were used to estimate the risk of clusters. Random forest was used to identified discriminative predictors in clusters, which were utilized to construct nomogram based on multivariable logistic regression in the discovery cohort. For easy clinical applications, we developed a bloodstream infections clustering (BSIC) score according to the nomogram. The results were validated in the validation cohort over a similar period.

**Results:**

A total of 360 patients in the discovery cohort and 310 patients in the validation cohort were included in statistical analyses. Based on baseline variables, two distinct clusters with differing prognostic outcomes were identified in the discovery cohort. Population in cluster 1 was 211 with a ICU mortality of 17.1%, while population in cluster 2 was 149 with an ICU mortality of 41.6% (*p* < 0.001). The survival analysis also revealed a higher risk of death for cluster 2 when compared with cluster 1 (hazard ratio: 2.31 [95% CI, 1.53 to 3.51], *p* < 0.001), which was confirmed in validation cohort. Four independent predictors (vasoconstrictor use before BSI, mechanical ventilation (MV) before BSI, Deep vein catheterization (DVC) before BSI, and antibiotic use before BSI) were identified and used to develop a nomogram. The nomogram and BSIC score showed good discrimination with AUC of 0.96.

**Conclusion:**

The developed score has potential applications in the identification of high-risk critically ill BSI patients.

**Supplementary Information:**

The online version contains supplementary material available at 10.1186/s12879-024-09203-5.

## Background

Bloodstream infections (BSI) are among the most serious infections causing sepsis or septic shock and are highly prevalent in hospitalized patients requiring intensive care, which can lead to prolonged hospital stays and high healthcare costs [1, 2]. Currently, the main therapeutic option for BSI patients is antimicrobial therapy, combined with optimal management of its consequences (such as shock, organs dysfunctions or metastatic suppurative complications) and surgical treatment (including debridement, abscess drainage, or removal of intravascular devices) when necessary. To achieve optimal clinical outcomes, timely and critical assessment of BSI patients are necessary to ensure prompt, effective, and targeted treatment [3]. However, the current standard of care mostly depends on blood culture-based diagnosis, which is often extremely slow [4]. Therefore, antimicrobial therapy is still empiric, targeting the most likely etiologic pathogens. Moreover, in recent years, there has been a rapid increase in the occurrence of antimicrobial-resistant pathogens in BSI, limiting treatment options and affecting the prognostic outcomes [3].

In the intensive care unit (ICU), BSI could be hospital-acquired, community-acquired or healthcare-associated. They are characterized by different epidemiology, risk factors, microbiology, sources, systemic responses and prognostic outcomes [5], increasing disease complexity and heterogeneity. Regarding heterogeneities, clinicians have tried to cluster critically ill patients into different sub-phenotypes based on clinically objective parameters [6–9]. To a certain degree, this improvement in recognition allows further understanding of disease classification and pathophysiology, potentially leading to precision treatments that reduce morbidity and mortality rates among critically ill patients. Thus, to identify patients at a high risk of BSI and to inform targeted/personalized management, there is an urgent need for better characterization of BSI phenotypes.

In this study, we hypothesized that applying a clustering approach to a database of BSI patients can help better characterize different BSI phenotypes, which may be of significance in constructing an easy-to-use nomogram for screening high-risk patients. To determine whether the developed model accurately predicts poor outcomes for BSI patients in ICU, we externally validated this model using an independent cohort.

## Methods

### Study design and participating cohorts

This retrospective observational study was conducted on two primary cohorts. For the development cohort, we collected data from patients who presented to the ICU at the First Affiliated Hospital of Xiamen University between January 2016 and December 2021. For external validation, we utilized data from an independent cohort at the First Hospital of Shanxi Medical University, that was retrospectively enrolled over a similar period.

The study was carried out according to the principles of the declaration of Helsinki and was approved by The Medical Ethics Committee of First Affiliated Hospital of Xiamen University (approval number: ky2021044) and First Hospital of Shanxi Medical University (approval number: 2021-K121) approved this study. Since the study was retrospectively conducted and no interventions were applied, the Ethics Committee of First Affiliated Hospital of Xiamen University and First Hospital of Shanxi Medical University approved the waiver of informed consent.

Patients were eligible for inclusion if they were aged ≥ 18 years and had a clinically positive blood culture for a bacterium or fungus obtained during their stay in the ICU [5]. The exclusion criteria was: incomplete core data, especially with regards to a lack of information on the specific treatment received before the diagnosis of BSI or its prognostic outcomes.

### Data collection

Research coordinators and board-certified ICU physicians collected demographic and clinical data from the patients using a case report form. They reviewed the electronic medical records and verified the final data. The following information was collected: demographic characteristics (age, gender, BMI, etc.), comorbidities, conditions before BSI (mechanical ventilation, deep vein catheterization, antibiotic use, vasoconstrictor use etc.), ICU complications (multiple organ dysfunction syndrome (MODS), acute respiratory distress syndrome (ARDS), septic shock, acute kidney injury (AKI) and disseminated intravascular coagulation (DIC)), outcomes (hospital stays, ICU stays and ICU mortality), primary site of infection, vital signs at baseline and results /from laboratory examinations. Vital signs at baseline and laboratory indicators, including inflammatory indicators and organ function damage indicators, were collected at the time point of blood sample collection. The baseline Sequential Organ Failure Assessment (SOFA) score and Pitt bacteremia score (Pitt score) were also calculated at the same timepoint [10, 11].

The main outcome was ICU mortality. The secondary outcomes were days of ICU stay, days of hospital stay, and ICU-associated complications such as MODS or septic shock.

### Statistical analysis

Categorical variables are presented as numbers (percentages), while continuous variables are presented as means ± SD or median (IQR) according to whether they were normally distributed or not. Statistical analyses were performed using R version 3.5.3 for Windows (http://www.r-project.org/). Categorical variables were compared by Chi-square or Fisher’s exact tests. Normally distributed variables were compared by the Student’s *t* test. The Mann-Whitney U test, a non-parametric test, was performed to compare variables that were not normally distributed. Based on all baseline variables, partitioning-based algorithms k-means was used to discover the groups of BSI phenotypes with different prognostic outcomes. Kaplan-Meier curves were constructed and compared using log-rank tests to validate the results of k-means. Univariate Cox regression analyses were used to estimate the risk of clusters.

In this study, random forest was developed to identify the predictors of the clusters. The selected predictors were subjected to multivariate logistic regression analysis, and a nomogram was developed. For easy clinical applications, a bloodstream infections clustering (BSIC) score was set based on the nomogram. The discriminative abilities of the nomogram were measured by area under the receiver operating characteristic curve (AUC). Sensitivity, specificity, positive predictive value (PPV) and negative predictive value (NPV) were proposed. The results were validated in the validation cohort of 310 adult BSI patients. We considered p values of less than 0·05 to be significant, and all tests were two-tailed.

## Results

### Patient characteristics

In the discovery cohort, 383 patients were initially recruited, of which 23 patients without complete data were excluded, leaving 360 patients who were eligible for analysis. For the validation cohort, 313 patients were initially enrolled, and 3 patients were excluded due to missing prognostic information. A final total of 310 patients were included in the validation cohort. Table 1 and S1 show the baseline characteristics (demographic characteristics, pre-existing conditions, primary sites of infection, vital signs at baseline, laboratory examination outcomes and specific treatments before BSI) as well as prognostic outcomes (ICU complication and outcomes) of patients in the discovery and validation cohorts. In the discovery cohort, the median age for the patients was 64 years, with 37.2% of the patients being female. The baseline SOFA scores and ICU mortality rate for this cohort were 8 and 27.2%, respectively. In the validation cohort, the median age and proportion of female patients were 62 years and 48.7%, respectively while the baseline SOFA scores and ICU mortality rate were 7 and 25.5%, respectively.

**Table 1:** Baseline characteristics and prognosis of patients in the discovery and validation cohorts

	Discovery cohort (*n* = 360)	Validation cohort (*n* = 310)
**Demographic characteristics**		
Age, years	64.00 [51.00, 74.00]	62.00 [47.00, 73.00]
Gender, female	134 (37.2)	151 (48.7)
BMI	22.66 [20.03, 25.39]	24.10 [21.22, 26.71]
From which department transfer to ICU		
Direct transfer from ED	177 (49.2)	92 (30.0)
Emergency room	23 (6.4)	4 (1.3)
Surgical department	132 (36.7)	166 (54.1)
Internal-medicine department	28 (7.8)	45 (14.7)
SOFA	8.00 [5.00, 11.00]	7.00 [5.00, 10.00]
Pitt bacteremia Score	3.00 [1.00, 6.00]	NA
**Pre-existing conditions**		
Cardiovascular disease	39 (10.8)	45 (14.5)
Cerebrovascular disease	34 (9.4)	38 (12.3)
Diabetes	82 (22.8)	64 (20.6)
Tumor	63 (17.5)	51 (16.5)
Chronic kidney diseases	15 (4.2)	15 (4.9)
Autoimmune disease	17 (4.7)	38 (12.3)
**Before bloodstream infection**		
hospital stays, days	4.00 [0.00, 13.25]	8.00 [2.00, 17.00]
ICU stays, days	1.00 [0.00, 8.00]	3.50 [1.00, 11.00]
Surgical operation	154 (42.8)	175 (56.5)
Mechanical ventilation	133 (36.9)	234 (75.5)
Deep vein catheterization	171 (47.5)	160 (51.6)
Antibiotic use	223 (61.9)	295 (95.2)
vasopressors use	110 (30.6)	130 (42.1)
**ICU complication**		
MODS	128 (35.6)	106 (34.2)
ARDS	164 (45.6)	88 (28.4)
Septic shock	141 (39.2)	158 (51.0)
AKI	112 (31.1)	150 (48.4)
DIC	39 (10.8)	42 (13.5)
**Outcomes**		
hospital stays, days	22.00 [12.00, 36.00]	27.00 [16.00, 49.00]
ICU stays, days	13.00 [7.75, 26.25]	15.00 [7.00, 29.00]
ICU mortality	98 (27.2)	79 (25.5)

Date was presented by mean ± standard deviation, n (%) or median (interquartile range)

BMI, body mass index; ICU, intensive care unit; ED, emergency department; SOFA, sequential organ failure assessment score; MODS, multiple organ dysfunction syndrome; ARDS, acute respiratory distress syndrome; AKI, acute kidney injury; DIC, disseminated intravascular coagulation

### Characteristics of clusters in the discovery cohort

**Fig. 1:**
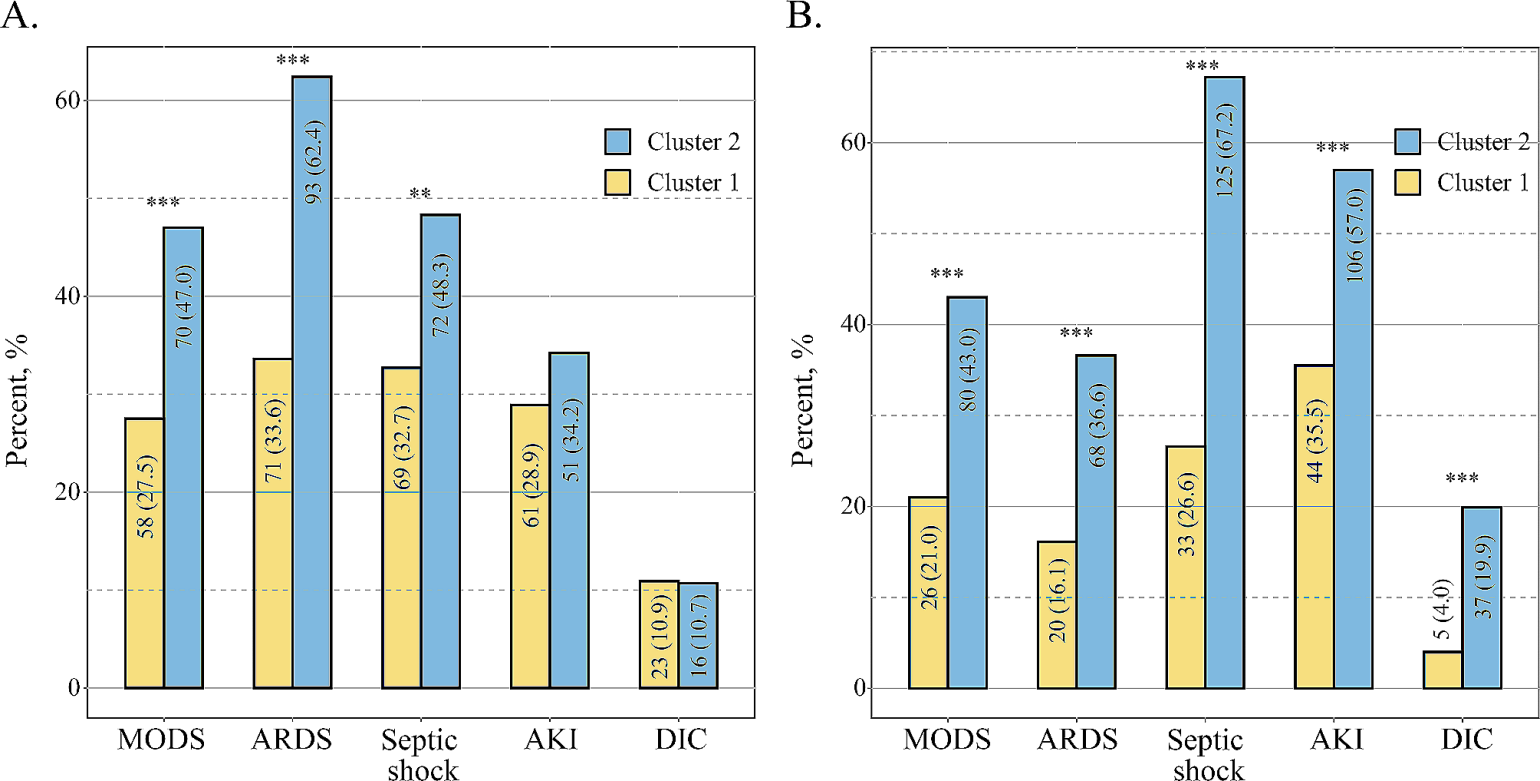
The organ dysfunction of patients in the discovery (**A**) and validation cohorts (**B**). Patients in cluster 2 had significantly more ICU complications (MODS, ARDS, septic shock, AKI and DIC). Abbreviation: MODS, multiple organ dysfunction syndrome; ARDS, acute respiratory distress syndrome; AKI, acute kidney injury; DIC, disseminated intravascular coagulation

Based on baseline variables, k-means analysis revealed two distinct clusters with differing prognostic outcomes. The clusters were well separated from one another, as shown S-Fig. 1. Patients in the two clusters had distinct baseline characteristics and prognostic outcomes (Table 2 and S-Table 2).

**Table 2:** Patient’s characteristics and prognosis difference in the clusters of discovery cohort

	Cluster 1 (*n* = 211)	Cluster 2 (*n* = 149)	*P* value
**Demographic characteristics**			
Age, years	64.00 [51.00, 73.00]	64.00 [50.00, 75.00]	0.450
Gender, female	89 (42.2)	45 (30.2)	0.027
BMI	22.49 [20.03, 25.37]	23.04 [19.95, 25.39]	0.837
From which department transfer to ICU			< 0.001
Direct transfer from ED	136 (64.5)	41 (27.5)	
Emergency room	13 (6.2)	10 (6.7)	
Surgical department	54 (25.6)	78 (52.3)	
Internal-medicine department	8 (3.8)	20 (13.4)	
SOFA	6.00 [4.00, 9.50]	9.00 [6.00, 12.00]	< 0.001
Pitt bacteremia Score	2.00 [0.00, 5.00]	5.00 [3.00, 6.00]	< 0.001
**Pre-existing conditions**			
Cardiovascular disease	21 (10.0)	18 (12.1)	0.640
Cerebrovascular disease	18 (8.5)	16 (10.7)	0.601
Diabetes	56 (26.5)	26 (17.4)	0.058
Tumor	27 (12.8)	36 (24.2)	0.008
Chronic kidney diseases	10 (4.7)	5 (3.4)	0.704
Autoimmune disease	11 (5.2)	6 (4.0)	0.787
**Before bloodstream infection**			
hospital stays, days	0.00 [0.00, 4.00]	13.00 [6.00, 21.00]	< 0.001
ICU stays, days	0.00 [0.00, 1.00]	7.00 [1.00, 12.00]	< 0.001
Surgical operation	58 (27.5)	96 (64.4)	< 0.001
Mechanical ventilation	16 (7.6)	117 (78.5)	< 0.001
Deep vein catheterization	38 (18.0)	133 (89.3)	< 0.001
Antibiotic use	78 (37.0)	145 (97.3)	< 0.001
vasopressors use	5 (2.4)	105 (70.5)	< 0.001
**ICU complication**			
MODS	58 (27.5)	70 (47.0)	< 0.001
ARDS	71 (33.6)	93 (62.4)	< 0.001
Septic shock	69 (32.7)	72 (48.3)	0.004
AKI	61 (28.9)	51 (34.2)	0.338
DIC	23 (10.9)	16 (10.7)	1.000
**Outcomes**			
hospital stays, days	15.00 [10.00, 27.00]	32.00 [21.00, 49.00]	< 0.001
ICU stays, days	11.00 [7.00, 18.50]	18.00 [9.00, 33.00]	< 0.001
ICU mortality	36 (17.1)	62 (41.6)	< 0.001

Date was presented by mean ± standard deviation, n (%) or median (interquartile range)

BMI, body mass index; ICU, intensive care unit; ED, emergency department; SOFA, sequential organ failure assessment score; MODS, multiple organ dysfunction syndrome; ARDS, acute respiratory distress syndrome; AKI, acute kidney injury; DIC, disseminated intravascular coagulation

Patients in cluster 1 (*n* = 211) were likely to have been directly transferred from the emergency department to ICU, and had less time in the hospital before BSI. These patients had milder organ dysfunctions (lower SOFA scores) and received fewer invasive treatments. The main primary sites of infection were the abdomen, followed by the urinary system, with fewer patients exhibiting pulmonary infections. Regarding prognosis, patients in cluster 1 were less likely to suffer from MODS, had lower incidences of ARDS and septic shock, and had lower ICU mortality rates (17.1%). Patients in cluster 2 (*n* = 149) had significantly higher SOFA scores, more ICU complications (MODS, ARDS, and septic shock) as shown in Table 2; Fig. 1A, and poorer prognostic outcomes (longer hospital and ICU stays, higher ICU mortality (41.6%)).

**Fig. 2:**
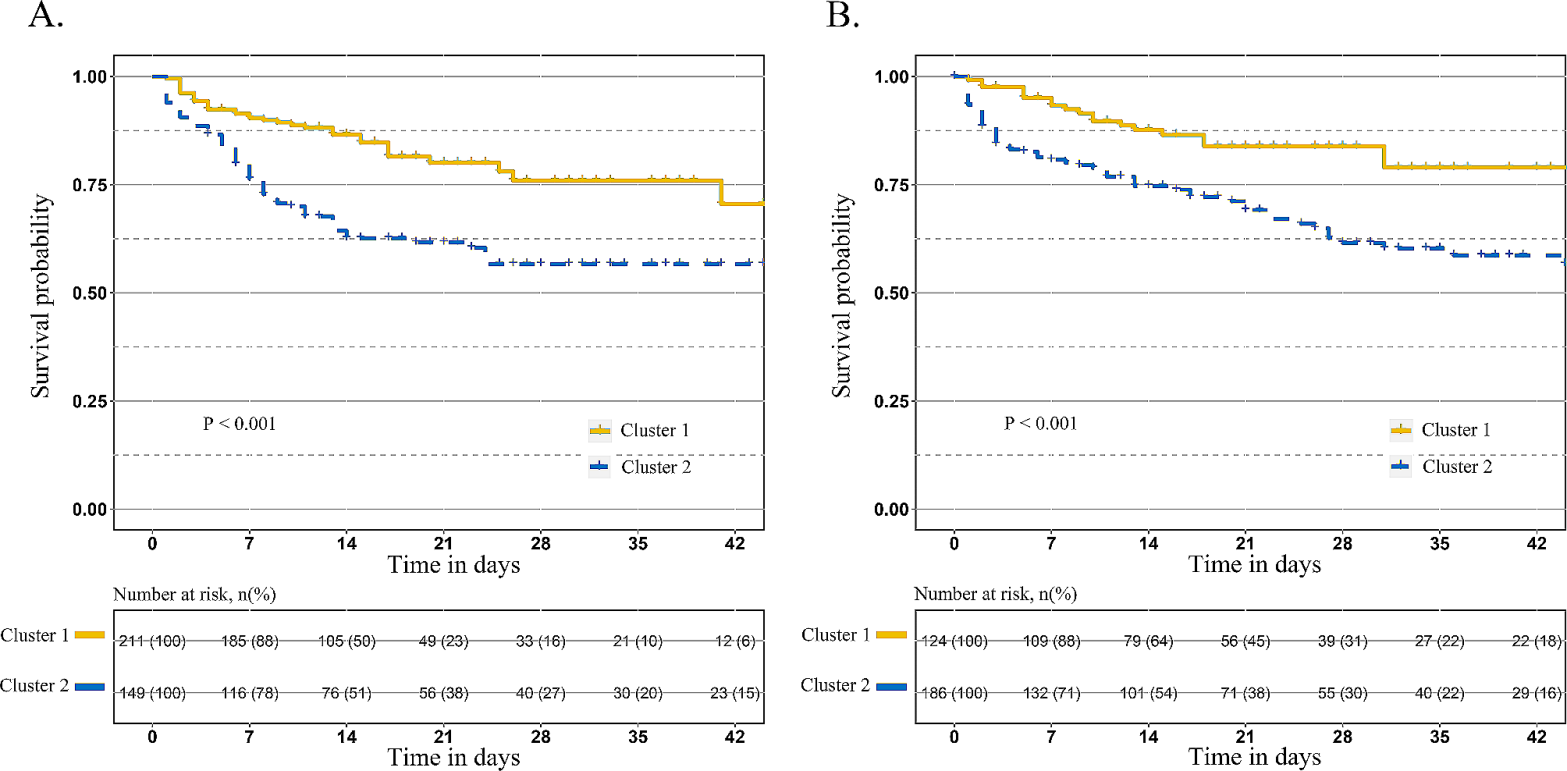
The Kaplan-Meier survival curves of patients in the discovery (**A**) and validation cohorts (**B**). The assumption of the proportional hazards was confirmed in Cox regression analysis. The Kaplan-Meier curve revealed a higher risk of death for cluster 2 patients, compared to cluster 1 (hazard ratio: 2.31 [95% CI, 1.53 to 3.51], *p* < 0.001, Fig. 2A). A higher proportion of patients in cluster 2 had been subjected to mechanical ventilation (117/149, 78.5%), deep vein catheterization (133/149, 89.3%), antibiotics (145/149, 97.3%) and vasoconstrictor agents (105/149, 70.5%) before the diagnosis of bloodstream infections

### Predicting the identified clusters using baseline variables

**Fig. 3:**
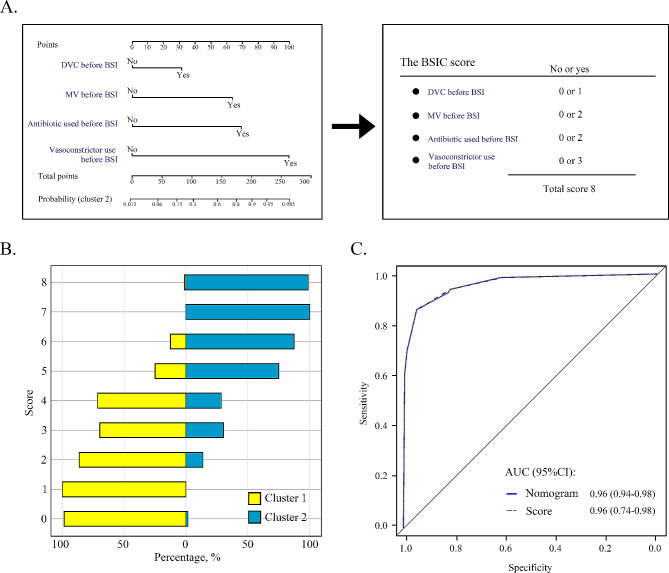
Predict the identified clusters in the patients with BSI. (**A**) Nomogram to predict the identified clusters. Points are assigned based on 4 baseline variable by drawing a line upward from the corresponding values to the “Points” line. The sum of these four points, plotted on the “Total points” line, corresponds to possibility of cluster 2. The bloodstream infections clustering (BSIC) score derives from the nomogram, allows the user to partition patients into 2 clusters by calculate total point. (**B**) Calibration plot of BSIC score. The proportion of patients attributed to cluster 2 increased with the score. (**C**) Receiver operating characteristic curves (ROC) for discrimination estimate. Abbreviation: DVC, Deep vein catheterization; MV, mechanical ventilation; BSI, bloodstream infection; AUC, area under the curve

Using the random forest, the four baseline variables (vasoconstrictor use before BSI, MV before BSI, DVC before BSI, and antibiotic used before BSI; Figure S2) were identified to predict the prognostic outcomes of the identified clusters in the discovery cohort. Then, we created a nomogram that integrated all four significant independent predictors. For easy clinical applications, based on the derived nomogram using only four baseline variables, we developed a bloodstream infections clustering (BSIC) score (Fig. 3A). Figure 3B shows adequate calibration of the score, as the proportion of patients attributed to cluster 2 increased with the score. The nomogram and BSIC score showed good discrimination with AUC of 0.96 (95%CI, 0.94 to 0.98 and 0.74–0.98, respectively, Fig. 3C). The optimal cut-off value of the score was 5. The accuracy, sensitivity and specificity according to this cut-off value were 91%, 86% and 95% respectively, with PPV of 92% and NPV of 90%.

### Validation of the BSIC score

The four baseline variables (vasoconstrictor use before BSI, MV before BSI, DVC before BSI, and antibiotic use before BSI) were used to predict cluster labels of the 310 BSI patients in the validation cohort with BSIC scores. In this study, 124 of 310 patients were assigned to cluster 1, while 186 patients were assigned to cluster 2. Patient’s baseline characteristics and prognostic differences between predicted clusters of the validation cohort are shown in S-Table 3. Consistent with findings from the discovery cohort, cluster 2 patients had higher SOFA scores, more ICU complications (MODS, ARDS, septic shock, AKI, DIC), and poorer prognostic outcomes (longer hospital stays and ICU stays, higher ICU mortality), compared with patients in cluster 1. And the results are also shown in (Figs. 1B and 2B). The Kaplan-Meier analysis revealed a high risk of death for cluster 2 patients, compared to cluster 1 (hazard ratio: 2.23 [95% CI, 1.34 to 3.71], *p* = 0.001).

**Table 3:** Patient’s prognosis difference between the predict clusters of validation cohort

	Cluster 1 (*n* = 124)	Cluster 2 (*n* = 186)	*P* value
**ICU complication**			
MODS	26 (21.0)	80 (43.0)	< 0.001
ARDS	20 (16.1)	68 (36.6)	< 0.001
Septic shock	33 (26.6)	125 (67.2)	< 0.001
AKI	44 (35.5)	106 (57.0)	< 0.001
DIC	5 (4.0)	37 (19.9)	< 0.001
**Outcomes**			
hospital stays, days	26.50 [16.00, 47.00]	27.50 [15.00, 52.00]	0.746
ICU stays, days	12.00 [5.00, 24.00]	18.00 [9.00, 35.75]	0.003
ICU mortality	20 (16.1)	59 (31.7)	0.003

Date was presented by mean ± standard deviation, n (%) or median (interquartile range)

ICU, intensive care unit; MODS, multiple organ dysfunction syndrome; ARDS, acute respiratory distress syndrome; AKI, acute kidney injury; DIC, disseminated intravascular coagulation

Moreover, the species of pathogens in the discovery and validation cohorts were as shown in Figure S3. *Escherichia coli*, *Klebsiella pneumoniae* and *Staphylococcu*s were the top 3 most common pathogens in the discovery cohort. In contrast, the most common pathogens in the validation cohort were *Staphylococcus, Candida* and *Klebsiella pneumoniae*, respectively.

## Discussion

In recent years, several scoring systems have been developed for stratifying the risk of patients with sepsis [12, 13], but not for patients with BSI. Therefore, we identified independent parameters from available data during ICU stay and constructed a novel score for predicting the prognostic outcomes of BSI, which may promote patient stratification and inform personalized interventions. The clustering approach combining baseline variables allowed us to characterize two distinct BSI phenotypes, the clinical profiles of which correspond to “good prognosis” patients (cluster 1) and “poor prognosis” patients (cluster 2). The established nomogram incorporated four factors: vasoconstrictor use before BSI, MV before BSI, DVC before BSI, and antibiotic use before BSI. The novel prediction instrument showed good discrimination as well as calibration, and was also successfully externally validated.

Initiation of vasopressors over the course of critical illness is usually due to profound and durable hypotension, which is independently associated with increased mortality [14]. Hence, vasoconstrictor use before BSI reveals illness severity. Nosocomial infections are an important determinant of the outcomes of ICU patients [15]. About 70% of nosocomial BSI in the ICU are secondary to other primary infections, and among them, catheter-related infections and respiratory tract infections are the leading sources of secondary episodes [5, 15–17]. Bloodstream infections are associated with prolonged mechanical ventilation and deep vein catheter indwelling [18, 19]. Our results are consistent with those of previous studies that showed that MV use before BSI as well as DVC interventions before BSI are two important predictors of poor prognostic outcomes for BSI patients. Additionally, inappropriate applications of antibiotics induce bacterial resistance [20], and antibiotic resistance in pathogens is a challenge that is associated with high morbidity and mortality rates [21]. Therefore, in our study, the antibiotics used before BSI were independent risk factors for its development.

The BSIC score has several strengths. A remarkable strength is its ease of use. The parameters obtained from the patient status in the early stages of the ICU stay are well-defined and easily obtainable. Our model was constructed using baseline variables and does not require information about the detailed laboratory examination. Another advantage of the BSIC score is that it was subjected to an independent external validation process and showed good discrimination, thereby minimizing interpretation variabilities and improving their generalizability as well as lending credibility to their usefulness in different BSI cohorts. In addition, it can help in identification of individuals at a high risk of BSI, for whom treatment with broad-spectrum antibiotics and effective early-stage rapid microbiological identification should be considered. Therefore, this score can be used as a screening tool to improve clinical care decisions for patients at a high risk of BSI.

This study has various limitations. First, the BSIC score was developed based on data retrospectively obtained at two-centre cohorts, and only patients with positive blood cultures were included in this study. Second, other valuable predictors may not have been included in our analysis. The presented scores will be improved as additional predictive variables are incorporated. Third, it was not determined whether interventions that are based on the BSIC score can improve the outcomes of BSI patients. Finally, the scores only apply to adult patients in ICU. Their purpose is to predict the prognosis of BSI patients during their ICU stay. Thus, further studies should be performed to determine whether this score can be extended to all BSI patients.

## Conclusions

In conclusion, using a clustering approach in a cohort of BSI patients, we identified two distinct BSI phenotypes that will help physicians to identify high-risk patients. Four independent predictors (vasoconstrictor use before BSI, MV before BSI, DVC before BSI, and antibiotic use before BSI) were identified. These predictors are readily available during the early ICU stay and are easy to obtain. They can be used to construct an easy-to-use score for predicting the prognosis of BSI patients in the ICU. The significance of this characterization in patient management and prognosis should be evaluated.

### Electronic supplementary material

Below is the link to the electronic supplementary material.

Supplementary Material 1

Supplementary Material 2

Supplementary Material 3

Supplementary Material 4

Supplementary Material 5

Supplementary Material 6

Supplementary Material 7

## Data Availability

The data that support the findings of this study are available from the corresponding author upon reasonable request.

## Abbreviations

BSI: Bloodstream infection
ICU: Intensive care unit
MV: Mechanical ventilation
DVC: Deep vein catheterization
BSIC: Bloodstream infectious clustering score
BMI: Body mass index
ICU: Intensive care unit
ED: Emergency department
SOFA: Sequential organ failure assessment score
MODS: Multiple organ dysfunction syndrome
ARDS: Acute respiratory distress syndrome
AKI: Acute kidney injury
DIC: Disseminated intravascular coagulation
SOFA: Sequential organ failure assessment
AUC: Area under the receiver operating characteristic curve
ROC: Receiver operating characteristic curve
PPV: Positive predictive value
NPV: Negative predictive value
