# Supplementary figures and images for: Bloodstream infection clusters for critically ill patients: analysis of two-center retrospective cohorts
Source: BMC Infect Dis. 2024 Mar 13;24:306. doi: 10.1186/s12879-024-09203-5 (PMC10935929; doi:10.1186/s12879-024-09203-5)

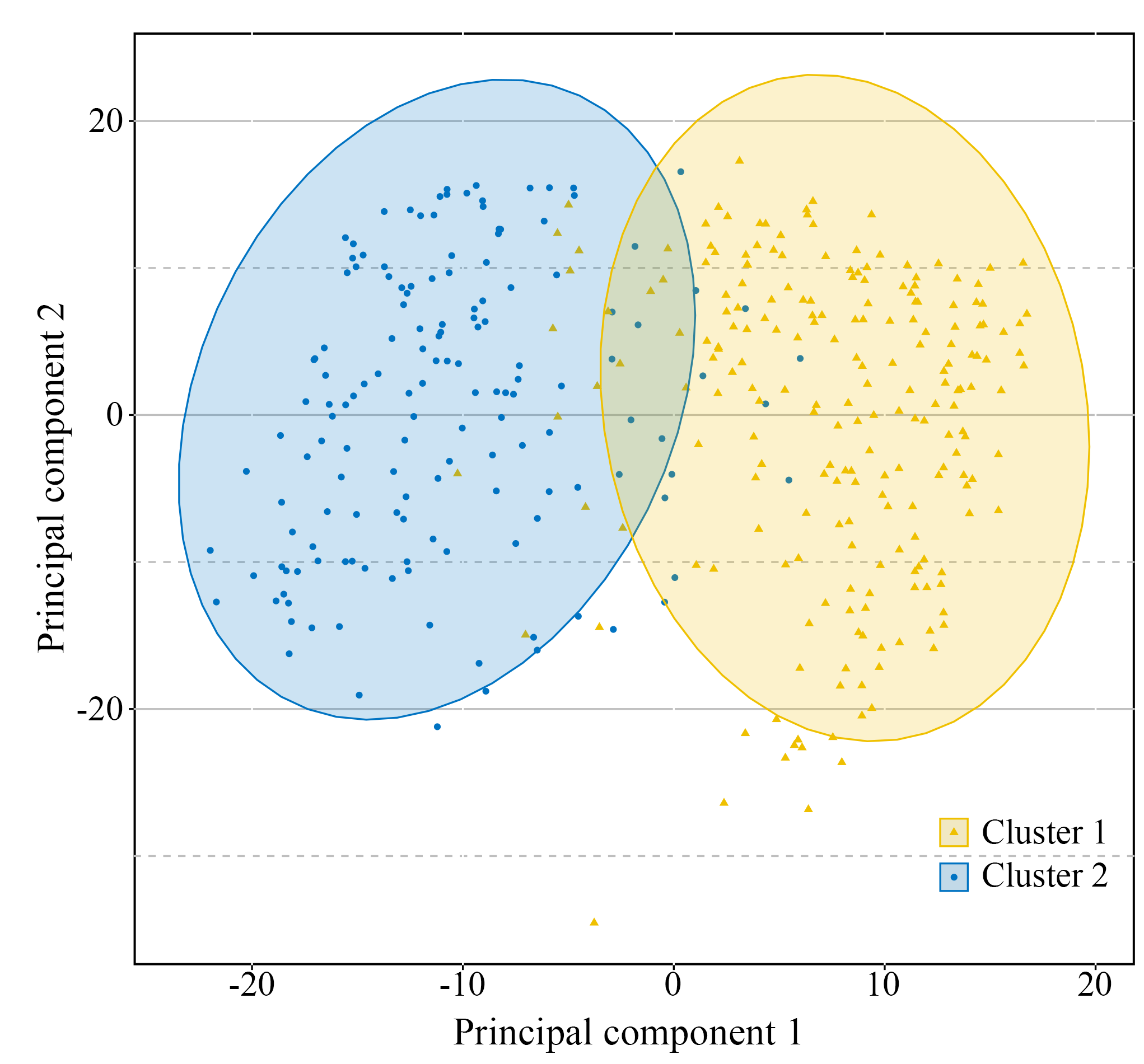

Supplement: Supplementary file 1 — Supplementary Material 1 [file 12879_2024_9203_MOESM1_ESM.tif]

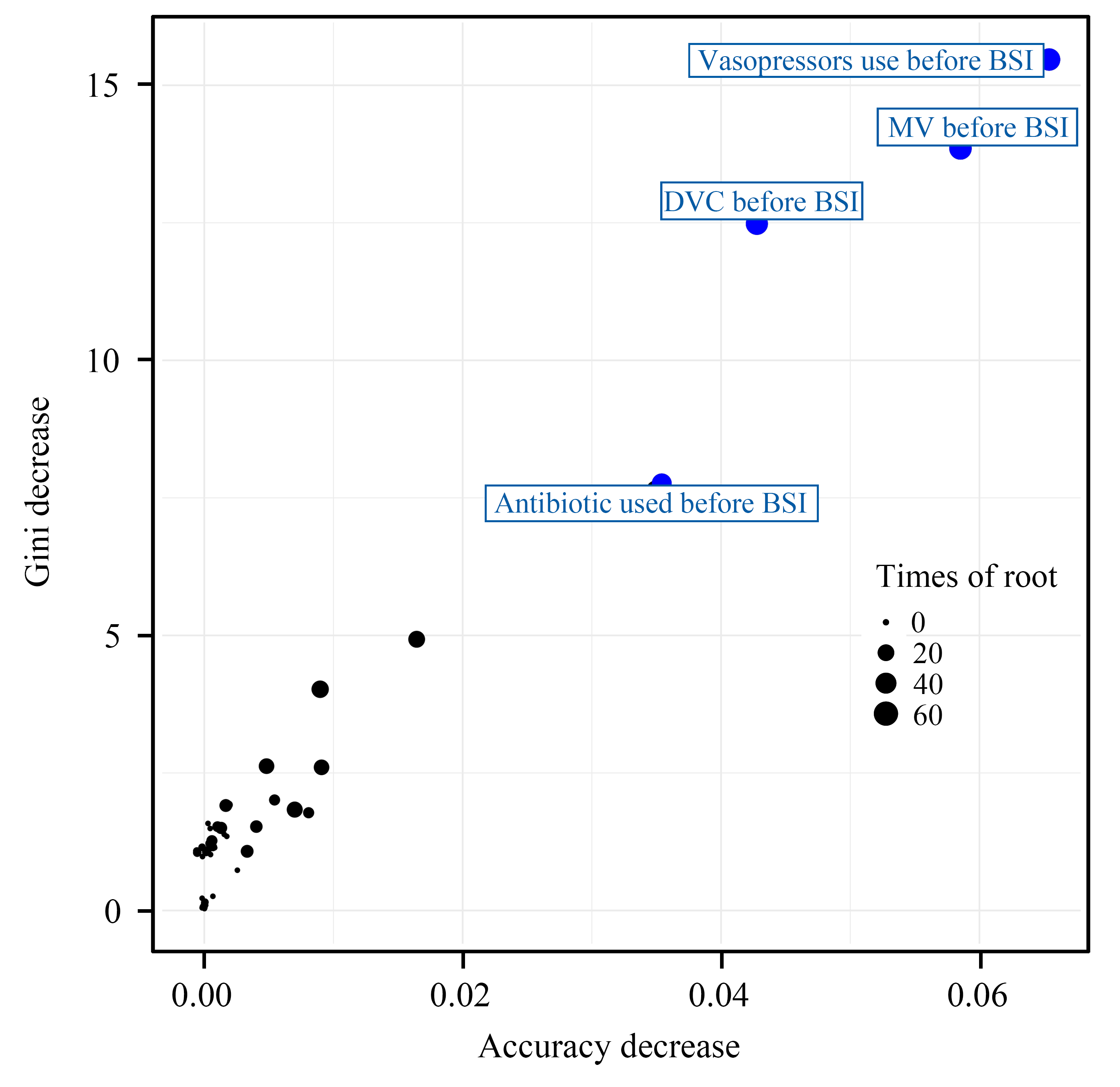

Supplement: Supplementary file 2 — Supplementary Material 2 [file 12879_2024_9203_MOESM2_ESM.tif]

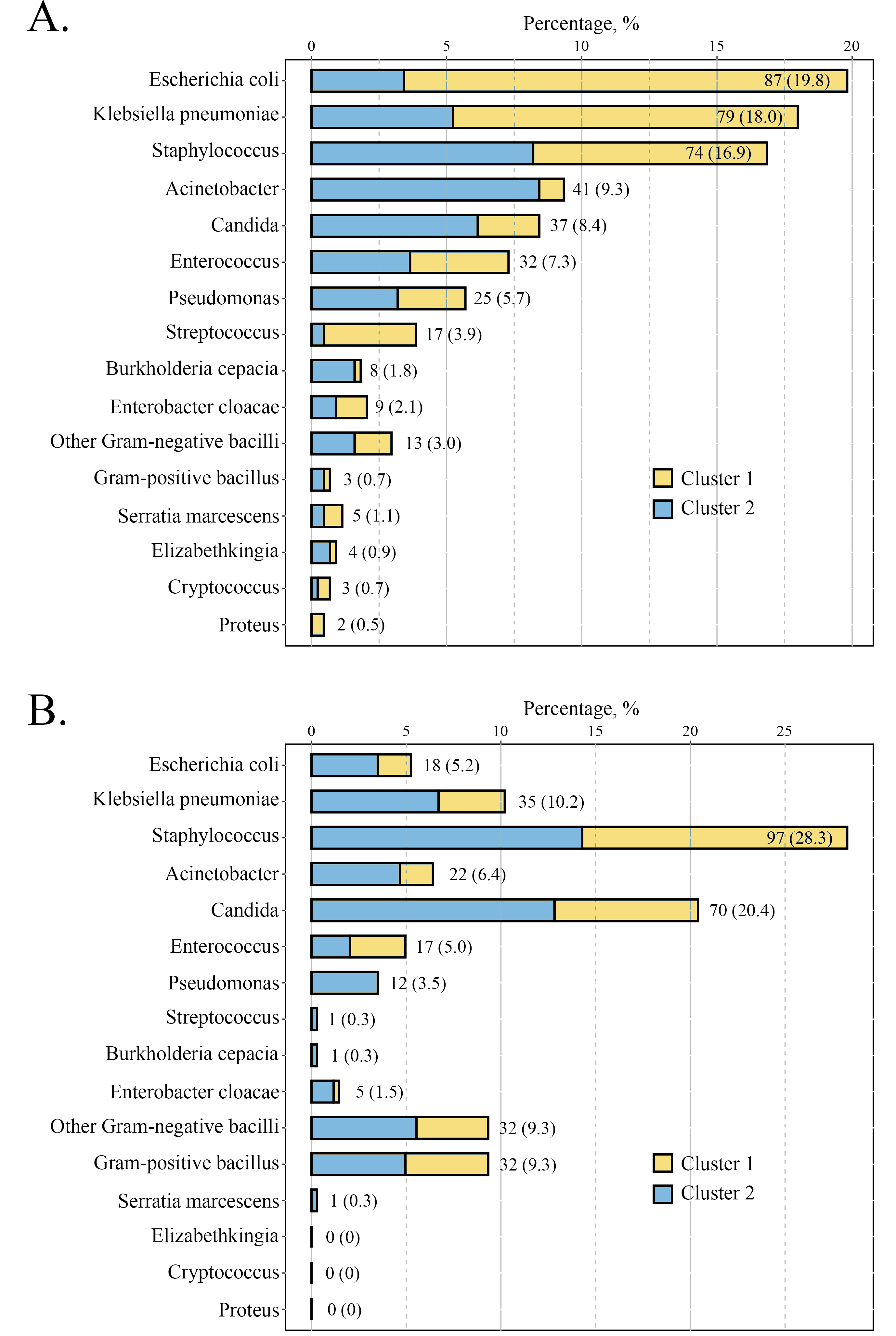

Supplement: Supplementary file 3 — Supplementary Material 3 [file 12879_2024_9203_MOESM3_ESM.tif]
