# Supplementary material for: Bloodstream infection clusters for critically ill patients: analysis of two-center retrospective cohorts
Source: BMC Infect Dis. 2024 Mar 13;24:306. doi: 10.1186/s12879-024-09203-5 (PMC10935929; doi:10.1186/s12879-024-09203-5)
Supplement: Supplementary file 4 — Supplementary Material 4 [file 12879_2024_9203_MOESM4_ESM.docx]

S-table 1: Supplementary baseline characteristics of patients in the discovery and validation cohorts

|  | Discovery cohort (n=360) | Validation cohort (n=310) |
| --- | --- | --- |
| **Primary site of infection** | | |
| Lung | 72 (20.0) | 110 (35.5) |
| Urinary system | 50 (13.9) | 28 (9.0) |
| Abdominal | 172 (47.8) | 116 (37.4) |
| Skin and soft tissue | 14 (3.9) | 22 (7.1) |
| Deep venous catheter | 23 (6.4) | 24 (7.7) |
| Others | 29 (8.1) | 10 (3.2) |
| **Vital signs at baseline** | | |
| Temperature, ℃ | 37.20 [36.60, 38.00] | 37.90 [37.00, 38.60] |
| Heart Rate, per minute | 103.00 [89.00, 118.00] | 96.50 [84.00, 112.00] |
| Respiratory rate, per minute | 21.00 [19.00, 25.00] | 20.00 [18.00, 24.00] |
| SAP, mmHg | 117.59 (22.39) | 121.90 (19.88) |
| DAP, mmHg | 65.59 (14.00) | 69.11 (12.39) |
| **Laboratory examination** | | |
| WBC, 10^9/L | 12.22 [8.23, 17.50] | 10.10 [6.90, 14.00] |
| PLT, 10^9/L | 144.50 [70.75, 234.75] | 142.00 [70.50, 215.00] |
| CRP, mg/L | 93.63 [51.08, 137.78] | NA |
| PCT, ng/ml | 6.21 [0.72, 40.00] | 1.57 [0.44, 11.22] |
| FIB, g/L | 4.18 [2.94, 5.59] | 3.90 [2.80, 4.85] |
| TBIL, umol/L | 17.60 [9.95, 37.40] | 18.90 [11.70, 39.38] |
| ALB, g/L | 29.80 [25.50, 33.00] | 29.10 [26.70, 32.32] |
| BUN, mmol/L | 10.29 [6.30, 14.72] | 9.15 [5.47, 13.91] |
| CRE, umol/L | 85.00 [58.00, 144.75] | 75.15 [52.02, 139.32] |
| PaO_2_/FiO_2_ | 252.00 [195.75, 338.75] | 200.50 [147.25, 290.00] |
| PH | 7.43 [7.39, 7.48] | 7.40 [7.36, 7.45] |
| Lactate, mmol/L | 1.70 [1.20, 3.12] | 2.50 [1.90, 3.40] |

Date was presented by mean ± standard deviation, n (%) or median (interquartile range).

SAP, systolic arterial pressure; DAP, diastolic arterial pressure; WBC, white blood cell count; PLT, platelet; CRT, C-reactive protein; PCT, procalcitonin; FIB, fibrionogen; TBIL, total bilirubin; ALB, albumin; BUN, blood urea nitrogen; CRE, creatinine; PaO2/FiO2, oxygenation index; PH, pondus hydrogenil.
