# Supplementary material for: Bloodstream infection clusters for critically ill patients: analysis of two-center retrospective cohorts
Source: BMC Infect Dis. 2024 Mar 13;24:306. doi: 10.1186/s12879-024-09203-5 (PMC10935929; doi:10.1186/s12879-024-09203-5)
Supplement: Supplementary file 5 — Supplementary Material 5 [file 12879_2024_9203_MOESM5_ESM.docx]

S-table 2: Supplementary baseline characteristics difference in the clusters of discovery cohort.

|  | Cluster 1 (n=211) | Cluster 2 (n=149) | P value |
| --- | --- | --- | --- |
| **Primary site of infection** | | | <0.001 |
| Lung | 30 (14.2) | 42 (28.2) |  |
| Urinary system | 41 (19.4) | 9 (6.0) |  |
| Abdominal | 108 (51.2) | 64 (43.0) |  |
| Skin and soft tissue | 9 (4.3) | 5 (3.4) |  |
| Deep venous catheter | 4 (1.9) | 19 (12.8) |  |
| Others | 19 (9.0) | 10 (6.7) |  |
| **Vital signs at baseline** | | |  |
| Temperature, ℃ | 37.10 [36.50, 38.00] | 37.20 [36.80, 38.00] | 0.225 |
| Heart Rate, per minute | 104.05 (20.77) | 103.64 (22.96) | 0.862 |
| Respiratory rate, per minute | 21.00 [19.00, 25.00] | 20.00 [18.00, 24.00] | 0.087 |
| SAP, mmHg | 115.09 (23.05) | 121.12 (20.98) | 0.012 |
| DAP, mmHg | 66.17 (13.89) | 64.77 (14.17) | 0.349 |
| **Laboratory examination** | | |  |
| WBC, 10^9/L | 12.63 [8.12, 17.10] | 12.08 [8.56, 18.21] | 0.496 |
| PLT, 10^9/L | 140.00 [68.00, 223.00] | 151.00 [71.00, 254.00] | 0.339 |
| CRP, mg/L | 97.27 [58.48, 144.00] | 82.84 [49.52, 123.85] | 0.064 |
| PCT, ng/ml | 17.60 [1.44, 57.99] | 2.49 [0.50, 9.50] | <0.001 |
| FIB, g/L | 4.64 [3.20, 5.91] | 3.70 [2.55, 4.92] | <0.001 |
| TBIL, umol/L | 16.70 [9.30, 35.60] | 19.12 [10.90, 39.00] | 0.242 |
| ALB, g/L | 28.70 [25.00, 32.15] | 31.50 [27.90, 34.50] | <0.001 |
| BUN, mmol/L | 9.51 [5.88, 14.06] | 11.46 [7.12, 15.49] | 0.024 |
| CRE, umol/L | 85.00 [61.50, 165.50] | 85.00 [55.00, 124.00] | 0.112 |
| PaO_2_/FiO_2_ | 266.00 [206.90, 346.50] | 232.00 [180.00, 325.00] | 0.025 |
| PH | 7.44 [7.39, 7.48] | 7.43 [7.38, 7.47] | 0.327 |
| Lactate, mmol/L | 1.70 [1.10, 3.45] | 1.70 [1.20, 2.80] | 0.996 |

Date was presented by mean ± standard deviation, n (%) or median (interquartile range).

SAP, systolic arterial pressure; DAP, diastolic arterial pressure; WBC, white blood cell count; PLT, platelet; CRT, C-reactive protein; PCT, procalcitonin; FIB, fibrionogen; TBIL, total bilirubin; ALB, albumin; BUN, blood urea nitrogen; CRE, creatinine; PaO2/FiO2,oxygenation index; PH, .pondus hydrogenil.
