# Supplementary material for: Bloodstream infection clusters for critically ill patients: analysis of two-center retrospective cohorts
Source: BMC Infect Dis. 2024 Mar 13;24:306. doi: 10.1186/s12879-024-09203-5 (PMC10935929; doi:10.1186/s12879-024-09203-5)
Supplement: Supplementary file 6 — Supplementary Material 6 [file 12879_2024_9203_MOESM6_ESM.docx]

S-table 3: Supplementary baseline characteristics difference in the predict clusters of validation cohort.

|  | Cluster 1 (n=124) | Cluster 2 (n=186) | P value |
| --- | --- | --- | --- |
| **Demographic characteristics** | | |  |
| Age, years | 62.00 [47.75, 73.00] | 62.50 [46.00, 72.75] | 0.677 |
| Gender, female | 61 (49.2) | 90 (48.4) | 0.981 |
| BMI | 24.35 [22.69, 26.62] | 23.50 [20.76, 26.71] | 0.101 |
| From which department transfer to ICU | | | 0.002 |
| Direct transfer from ED | 22 (18.0) | 70 (37.8) | 22 (18.0) |
| Emergency room | 1 (0.8) | 3 (1.6) | 1 (0.8) |
| Surgical department | 77 (63.1) | 89 (48.1) | 77 (63.1) |
| Internal-medicine department | 22 (18.0) | 23 (12.4) | 22 (18.0) |
| SOFA | 5.00 [4.00, 7.00] | 9.00 [6.00, 12.00] | <0.001 |
| **Pre-existing conditions** | | |  |
| Cardiovascular disease | 17 (13.7) | 28 (15.1) | 0.869 |
| Cerebrovascular disease | 19 (15.3) | 19 (10.2) | 0.243 |
| Diabetes | 31 (25.0) | 33 (17.7) | 0.160 |
| Tumor | 19 (15.3) | 32 (17.2) | 0.778 |
| Chronic kidney diseases | 4 (3.3) | 11 (5.9) | 0.421 |
| Autoimmune disease | 9 (7.3) | 29 (15.6) | 0.044 |
| **Before bloodstream infection** | | |  |
| hospital stays, days | 5.00 [1.00, 14.00] | 10.00 [3.00, 20.00] | 0.005 |
| ICU stays, days | 2.00 [0.00, 6.00] | 5.00 [1.00, 14.75] | <0.001 |
| Surgical operation | 80 (64.5) | 95 (51.1) | 0.026 |
| Mechanical ventilation | 67 (54.0) | 167 (89.8) | <0.001 |
| Deep vein catheterization | 22 (17.7) | 138 (74.2) | <0.001 |
| Antibiotic use | 112 (90.3) | 183 (98.4) | 0.003 |
| vasopressors use | 1 (0.8) | 129 (69.4) | <0.001 |
| **Primary site of infection** | | | 0.200 |
| Lung | 38 (30.6) | 72 (38.7) |  |
| Urinary system | 14 (11.3) | 14 (7.5) |  |
| Abdominal | 43 (34.7) | 73 (39.2) |  |
| Skin and soft tissue | 13 (10.5) | 9 (4.8) |  |
| Deep venous catheter | 12 (9.7) | 12 (6.5) |  |
| Others | 4 (3.2) | 6 (3.2) |  |
| **Vital signs at baseline** | | |  |
| Temperature, ℃ | 37.80 [37.00, 38.50] | 38.00 [37.02, 38.70] | 0.395 |
| Heart Rate, per minute | 92.00 [81.75, 106.00] | 100.00 [85.00, 118.00] | 0.014 |
| Respiratory rate, per minute | 20.00 [18.00, 23.00] | 20.00 [17.25, 25.00] | 0.638 |
| SAP, mmHg | 126.59 (18.52) | 118.78 (20.19) | 0.001 |
| DAP, mmHg | 71.80 (11.27) | 67.32 (12.80) | 0.002 |
| **Laboratory examination** | | |  |
| WBC, 10^9/L | 10.10 [7.18, 14.05] | 10.05 [6.73, 14.00] | 0.596 |
| PLT, 10^9/L | 160.00 [95.75, 228.75] | 129.00 [60.50, 202.75] | 0.008 |
| PCT, ng/ml | 0.99 [0.34, 8.33] | 2.05 [0.58, 12.26] | 0.007 |
| FIB, g/L | 4.13 [3.30, 5.58] | 3.53 [2.44, 4.57] | 0.001 |
| TBIL, umol/L | 17.60 [10.75, 32.15] | 19.70 [12.10, 45.90] | 0.107 |
| ALB, g/L | 29.50 [26.60, 33.50] | 28.90 [26.80, 31.50] | 0.054 |
| BUN, mmol/L | 7.55 [4.61, 12.23] | 9.96 [6.37, 16.06] | 0.002 |
| CRE, umol/L | 65.75 [51.77, 97.00] | 84.90 [53.08, 155.93] | 0.004 |
| PaO_2_/FiO_2_ | 218.50 [160.00, 302.50] | 194.50 [141.25, 275.00] | 0.011 |
| PH | 7.41 [7.37, 7.45] | 7.40 [7.35, 7.44] | 0.046 |
| Lactate, mmol/L | 2.30 [1.70, 3.05] | 2.55 [1.90, 3.77] | 0.029 |

Date was presented by mean ± standard deviation, n (%) or median (interquartile range).

BMI, body mass index; ICU, intensive care unit; ED, emergency department; SOFA, sequential organ failure assessment score; SAP, systolic arterial pressure; DAP, diastolic arterial pressure; WBC, white blood cell count; PLT, platelet; CRT, C-reactive protein; PCT, procalcitonin; FIB, fibrionogen; TBIL, total bilirubin; ALB, albumin; BUN, blood urea nitrogen; CRE, creatinine; PaO2/FiO2, oxygenation index; PH, pondus hydrogenil.
