# Supplementary material for: Bloodstream infection clusters for critically ill patients: analysis of two-center retrospective cohorts
Source: BMC Infect Dis. 2024 Mar 13;24:306. doi: 10.1186/s12879-024-09203-5 (PMC10935929; doi:10.1186/s12879-024-09203-5)
Supplement: Supplementary file 7 — Supplementary Material 7 [file 12879_2024_9203_MOESM7_ESM.docx]

**S-Figure 1** Two clusters in patients with Bloodstream infections

**S-Figure 2** The plot of variables importance base on random forest

**S-Figure 3** The species of pathogens in the discovery and validation cohorts
